# Impact of sport disciplines on gut microbiota in elite athletes: evidence from Taekwondo and diving athletes

**DOI:** 10.3389/fmicb.2026.1770499

**Published:** 2026-05-07

**Authors:** Xuelian Chen, Lihui Xie, Bing Li

**Affiliations:** School of Physical Education, Southwest University, Chongqing, China

**Keywords:** athletes' gut microbiota, diving athletes, gut microbiota, physical exercise, Taekwondo

## Abstract

**Objective:**

Gut microbiota is closely associated with athletic performance, but differences between sport disciplines have rarely been reported. This study collected gut microbiota samples from athletes across different sport disciplines to further elucidate sport-specific effects on gut microbiota, thereby providing empirical evidence for the “exercise-gut microbiota” relationship within specialized athletic domains.

**Methods:**

Gut microbiota from 27 professional Taekwondo athletes and 11 diving athletes underwent high-throughput sequencing analysis.

**Results:**

(1) No significant difference existed in gut microbiota α-diversity between Taekwondo and diving athletes; (2) No significant differences were observed in gut microbiota structure (β-diversity) between Taekwondo athletes and diving athletes; (3) Intergroup differences existed in the gut microbiota of Taekwondo athletes vs. diving athletes; (4) Regarding KEGG pathways of gut microbiota between Taekwondo and diving athletes, 12 metabolic pathways showed significant differences. Specifically, lipid IVA biosynthesis and Kdo transfer to lipid IVA III pathways were lower in Taekwondo athletes than in diving athletes, whereas pathways including glycine biosynthesis of tetrapyrrole II, and inositol, chiro-inositol, and scyllo-inositol degradation were higher.

**Conclusion:**

Taekwondo athletes and diving athletes exhibit distinct differences in gut microbiota composition and multiple metabolic pathways, but show no significant difference in diversity.

## 1 Introduction

The collective community of bacterial microorganisms residing in the gastrointestinal tract is referred to as gut microbiota. Human gut microbiota primarily comprises four major phyla: Firmicutes and Bacteroidetes, which collectively account for approximately 90% of the gut microbiota, followed by Actinobacteria and Proteobacteria (Arumugam et al., 2011). Appropriate exercise modulates the composition and abundance of gut microbiota, exerting positive health effects such as improving intestinal mucosal permeability, alleviating exercise-induced inflammation, and enhancing body composition (Mohr et al., 2020; Sohail et al., 2019). It also induces beneficial alterations in gut microbiota composition and metabolites, thereby promoting host health (Clauss et al., 2021). Primary metabolites of gut microbiota include short-chain fatty acids (SCFAs) and branched-chain fatty acids (BCFAs) (Hooper and Gordon, 2001). SCFAs stimulate intestinal enteroendocrine cells to secrete gut-derived serotonin, influencing the excitability of intestinal afferent nerves (O'Donovan et al., 2020). Branched-chain amino acids can provide energy reserves for prolonged endurance exercise, promote muscle protein synthesis, and regulate peripheral mechanisms triggering exercise-induced fatigue, thereby delaying its onset; furthermore, post-exercise BCAA supplementation facilitates recovery from exercise-induced fatigue (Sakurai et al., 2018).

Exercise training is closely associated with the gut microbiota. Studies have shown that the relationship between exercise and the gut microbiota has garnered significant attention from researchers. Exercise training can promote human health through its positive effects on the gut microbiota. Furthermore, athletes from various sport disciplines have demonstrated relevant differences in gut microbiota composition, including wrestling (Fu et al., 2024), martial arts (Liang et al., 2019), swimming (Bielik et al., 2022), and long-distance running (Kulecka et al., 2020). Athletes from various sports disciplines have demonstrated relevant differences in gut microbiota composition. Current research indicates that athletes exhibit greater gut microbiota richness compared to non-athlete populations (Marttinen et al., 2020). Athletes show higher variability and abundance of Firmicutes than non-athletes, potentially attributable to differential exercise volumes, suggesting physical activity may determine gut microbiota composition (Dorelli et al., 2021). Recent studies reveal athletic-level differences in athletes' gut microbiota. Analysis of 19 Chinese female professional rowers (including elite and non-elite athletes) showed significantly higher mean relative abundance of Firmicutes in adult elite athletes and young elite athletes vs. young non-elite athletes, whereas Bacteroidetes exhibited significantly higher mean relative abundance in young non-elite athletes compared to adult and young elite athletes (Han et al., 2020).

Currently, research on whether gut microbiota differences exist among athletes from different sport disciplines remains rarely investigated. Therefore, this study examined elite Taekwondo and diving athletes. Using high-throughput sequencing of gut microbiota, we analyzed compositional differences to identify distinct functional characteristics between these two groups. For instance, Taekwondo athletes may enrich bacterial genera associated with rapid energy metabolism, while diving athletes may require microbiota balance maintaining intestinal barrier integrity and metabolic homeostasis. This investigation clarifies whether gut microbiota exhibits sport-specific differences, providing a new theoretical basis and practical approaches for enhancing competitive performance and training adaptability across different sport disciplines.

## 2 Subjects and methods

### 2.1 Subjects

This study included 27 elite Taekwondo athletes and 11 elite diving athletes, all ranked National First-Class Athletes or higher. All participants had maintained systematic specialized training for at least 4 years, with weekly training exceeding 20 h at moderate to high intensity per session, alongside fixed dietary routines. Exclusion criteria: athletes with injuries, unstable diets, gastrointestinal diseases, or occurrence of diarrhea. During the experiment, subjects refrained from taking any probiotic preparations (e.g., yogurt), antibiotics, or other medications that could interfere with testing. The athletes' basic information is presented in Table 1.

**Table 1:** Basic information of athletes.

Sport disciplines	Age (years)	Height (cm)	Weight (kg)	Training experience (years)
Taekwondo athletes	20.8 ± 1.8	172.3 ± 9.8	60.7 ± 9.6	9.3 ± 2.9
Diving athletes	10.3 ± 1.6	140.0 ± 12.4	34.4 ± 9.9	6.0 ± 3.6

### 2.2 Research methods

#### 2.2.1 Sample collection and transportation

Fecal sample collection and DNA extraction: Experimental personnel collected fecal samples from subjects daily between 6:00 and 7:00 AM. Samples were stored at −80 °C within 3 h post-collection. All collected samples were sent to Shanghai Majorbio Bio-pharm Technology Co., Ltd. for sequencing.

DNA extraction was performed using the FastDNA Stool Mini Kit (Qiagen, California, USA), with bacterial DNA concentration measured by NanoDrop 2000 spectrophotometer (Thermo Scientific, USA).

High-throughput sequencing: The bacterial communities in fecal samples were analyzed using Illumina-MiSeq high-throughput sequencing technology. The V3 and V4 regions of 16S rDNA were selected for PCR amplification and product purification, followed by construction of PE libraries and sequencing.

OTU clustering: Non-redundant gut microbiota sequences were clustered at 97% similarity threshold to generate OTU tables.

Diversity analysis: This study employed the Shannon index to analyze the α-diversity of gut microbiota in samples from different sport disciplines.

Analysis of microbial species composition: Taxonomic analysis revealed the species composition of athletes across various classification levels for different sport disciplines. Structural differences in gut microbiota among athletes of different disciplines were compared using Principal Coordinates Analysis (PCoA analysis). Species differences in gut microbiota among athletes of different disciplines were identified through LEfSe multilevel species discriminant analysis.

### 2.3 Statistical analysis

For gut microbiota data analysis, α-diversity indices were calculated using Mothur software. Intergroup differences in gut microbiota α-diversity among athletes from different sports were analyzed using the Wilcoxon rank-sum test. Structural differences in gut microbiota among athletes from different sports were compared using Principal Coordinates Analysis (PCoA) based on weighted UniFrac distance. Species-level differences were assessed through LEfSe multilevel species discriminant analysis. The significance threshold was set at *P* < 0.05, with *P* < 0.01 indicating highly significant differences.

## 3 Results

### 3.1 Comparison of gut microbiota α-diversity among athletes from different sports

**Figure 1:**
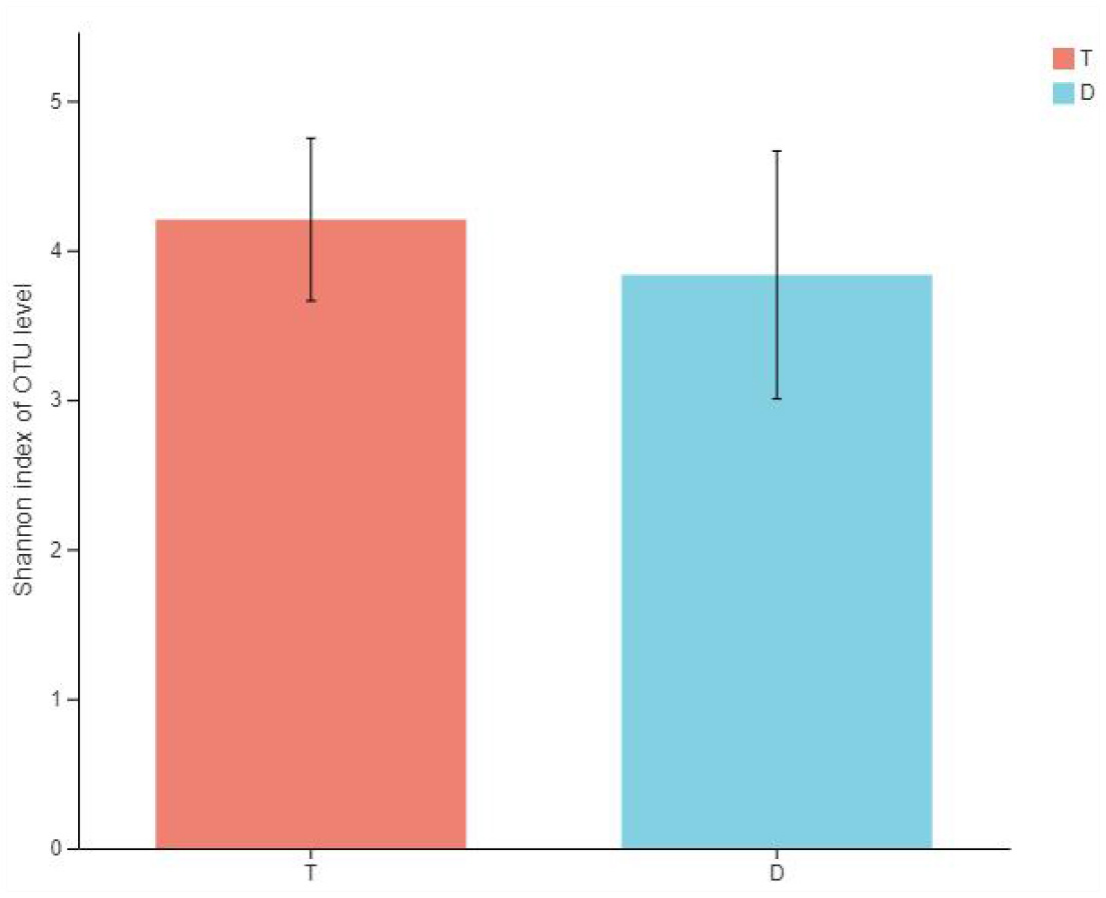
Comparison of gut microbiota α-diversity differences among athletes from different sports. *Note T* represents Taekwondo athletes, *D* represents diving athletes (the same applies below).

This study compared gut microbiota diversity between Taekwondo athletes and diving athletes using the Shannon index. Intergroup differences were evaluated with the Wilcoxon rank-sum test. As shown in Figure 1, no significant differences in gut microbiota α-diversity were observed between Taekwondo athletes and diving athletes.

### 3.2 Analysis of gut microbiota structural differences

**Figure 2:**
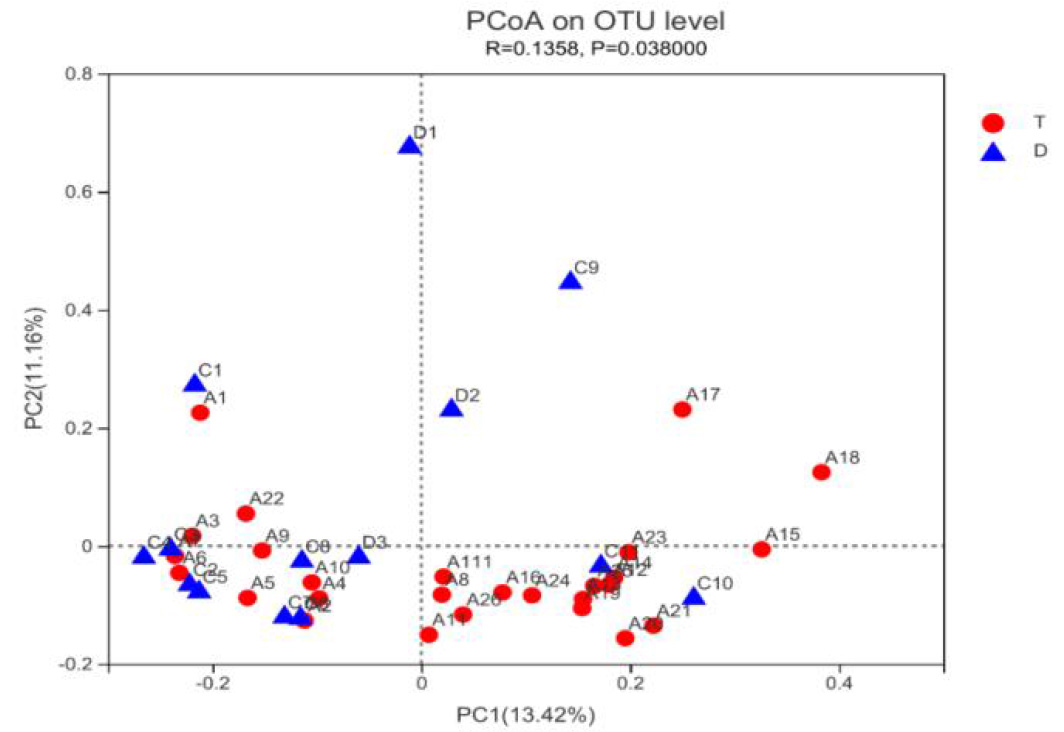
Beta diversity analysis of gut microbiota species in athletes.

Using PCoA analysis (Principal co-ordinates analysis) based on unweighted UniFrac distances, we examined structural differences (β-diversity) in gut microbiota between Taekwondo and diving athletes. As shown in Figure 2, the PCoA results indicate no significant difference in gut microbiota structure between the two athlete groups.

### 3.3 Analysis of gut microbiota species differences between Taekwondo and diving athletes

**Figure 3:**
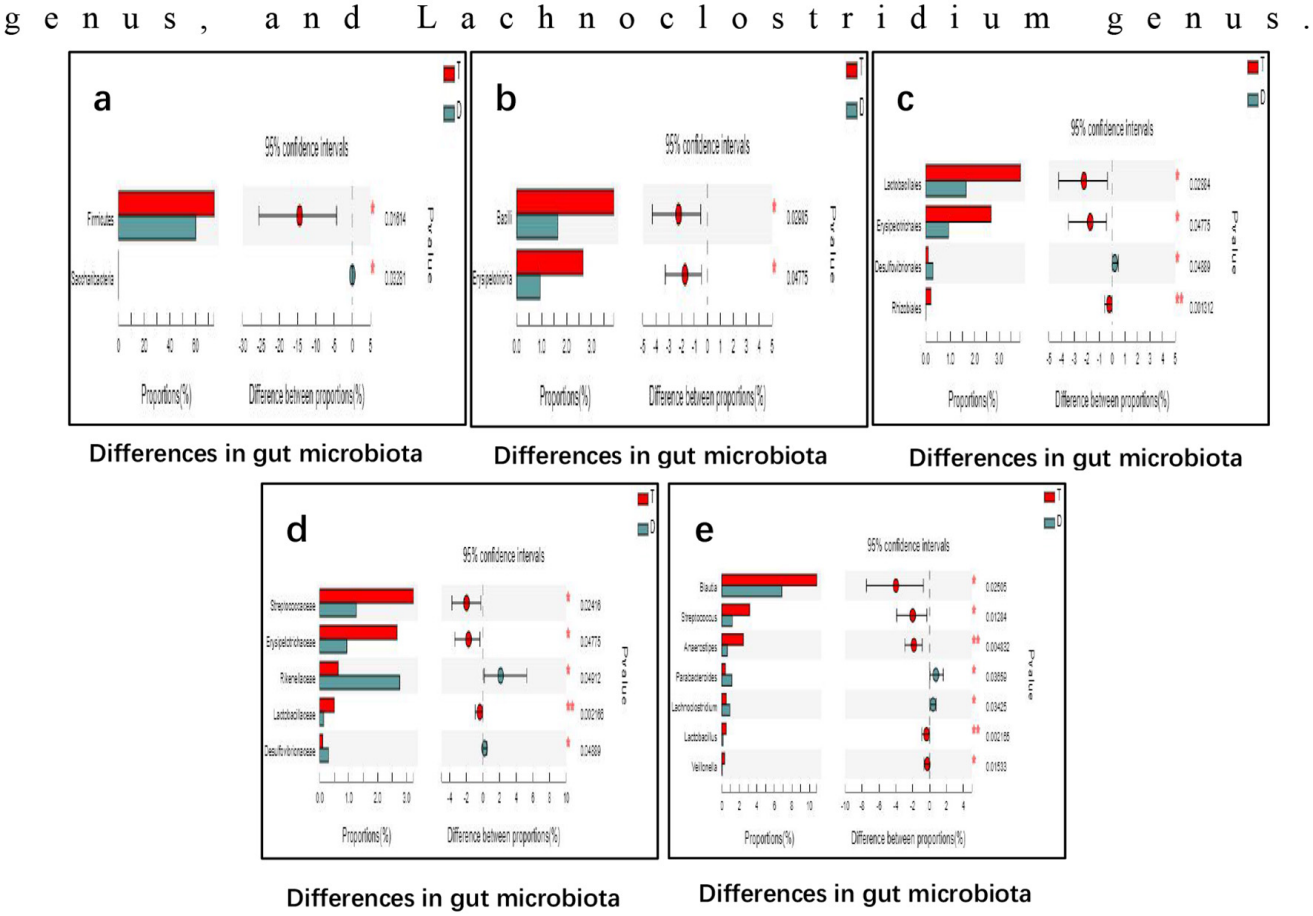
Differences in gut microbiota composition between Taekwondo and diving athletes at different taxonomic levels: **(a)** phylum, **(b)** class, **(c)** order, **(d)** family, and **(e)** genus. Bars on the left of each panel show the relative proportions (%) of each taxon in the two groups, and the right side shows the differences between proportions with 95% confidence intervals and corresponding *p*-values.

As shown in Figure 3, intergroup difference tests on gut microbiota revealed significantly higher abundances of Firmicutes (phylum), Bacilli (class), Erysipelotrichia (class), Lactobacillales (order), Erysipelotrichales (order), Rhizobiales (order), Streptococcaceae (family), Erysipelotrichaceae (family), Lactobacillaceae (family), Blautia genus, Streptococcus genus, Anaerostipes genus, Lactobacillus genus, and Veillonella genus in Taekwondo athletes compared to diving athletes; conversely, diving athletes exhibited significantly higher abundances of Saccharibacteria (phylum), Desulfovibrionales (order), Rikenellaceae (family), Desulfovibrionaceae (family), Parabacteroides genus, and Lachnoclostridium genus.

### 3.4 Functional differences in gut microbiota between Taekwondo and diving athletes

**Figure 4:**
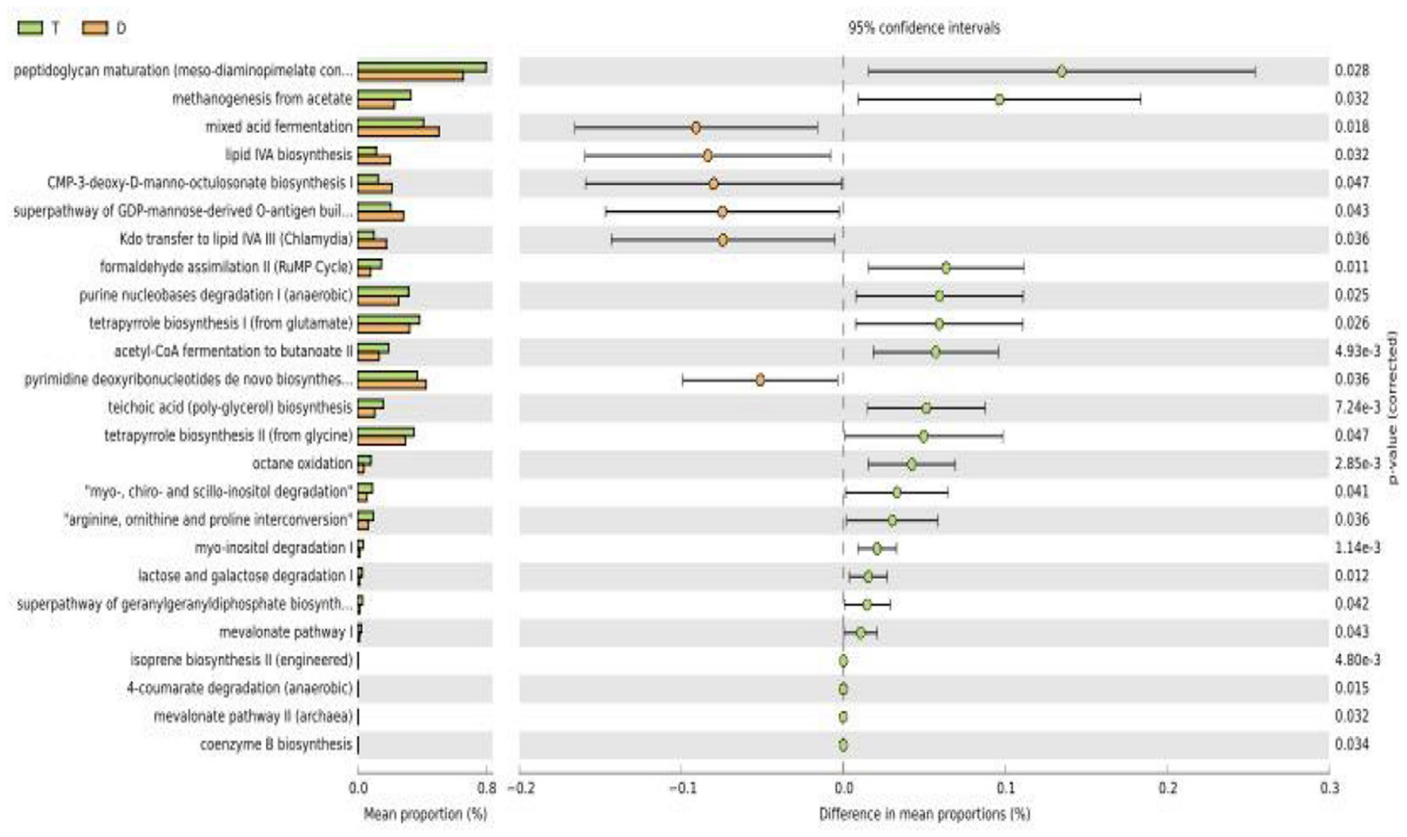
Functional differences in gut microbiota between Taekwondo and diving athletes.

The KEGG pathways between gut microbiota of Taekwondo athletes and diving athletes were predicted using PICRUSt2.0. As shown in Figure 4, we identified a total of 17 metabolic pathways, 12 of which exhibited significant differences. Figure 4 demonstrates that pathways including Peptidoglycan maturation (meso-diaminopimelate-containing), Methanogenesis from acetate, Formaldehyde assimilation II (RuMP Cycle), Purine nucleobases degradation I (anaerobic), Tetrapyrrole biosynthesis II (from glycine), myo-, chiro- and scyllo-inositol degradation, and arginine, ornithine and proline interconversion I were significantly elevated in Taekwondo athletes compared to diving athletes. Conversely, Mixed acid fermentation, Lipid IVA biosynthesis, CMP-3-deoxy-D-manno-octulosonate biosynthesis, Super pathway of GDP-mannose-derived O-antigen build, and Kdo transfer to lipid IVA III (Chlamydia) showed significantly lower activity in Taekwondo athletes.

## 4 Discussion

A notable limitation of this study is the significant differences in age, training duration, and body composition between the Taekwondo and diving athlete groups. As shown in Table 1, these disparities complicate the attribution of the observed microbiota differences solely to the “sport specialization” factor. It is likely that the observed differences result from a combination of factors, including the specific training regimens, the athletes' developmental stages, long-term training adaptations, and potential differences in dietary structures. However, these background differences mirror the “real-world” context of elite athlete selection and development across various sports. For example, in diving, athletes must rapidly master complex skills during the early stages of physical development, which may explain why it is not uncommon for Olympic champions, such as Quan Hongchan, to win titles as early as 14 years old. In contrast, combat sports like Taekwondo rely more heavily on strength and physical conditioning that develops in adulthood. Thus, the aim of this study was not to isolate a pure “sport specialization effect,” but rather to describe the specific gut microbiota profiles of elite athletes within the context of their respective training backgrounds. Despite potential confounding factors, the significant differences in microbiota structure and predicted functions between the two groups strongly suggest that “long-term specialized training” and “specific developmental stages” may jointly shape a gut microbiota that aligns with the athletes' physiological needs. Future studies should include more homogeneous participant groups (e.g., athletes of the same age group but from different sports) to further elucidate the independent contributions of these factors.

### 4.1 Comparison of gut microbiota α-diversity and β-diversity among athletes from different sports

Gut microbiota diversity serves as a potential biomarker and health indicator, playing a crucial role in maintaining intestinal homeostasis and promoting overall health (Shanahan, 2010). Its reduction is closely associated with various diseases such as autism spectrum disorder (Kang et al., 2013), gastrointestinal disorders (including Inflammatory Bowel Disease (IBD), recurrent Clostridium difficile-induced diarrhea), and obesity-related inflammatory characteristics (Ott et al., 2004), while increased diversity is associated with improved health indicators in the elderly (Claesson et al., 2012; Le Chatelier et al., 2013). Growing experimental evidence indicates that exercise increases gut microbiota diversity in the general population. Single sessions of moderate-intensity acute exercise (Clarke et al., 2014) and prolonged endurance training (Barton et al., 2021) both significantly enhance gut microbiota α-diversity in subjects. Furthermore, athletes exhibit higher gut microbiota α-diversity than non-athletes. A cross-sectional study revealed that professional rugby players have significantly higher gut microbiota α-diversity than sedentary individuals. This α-diversity positively correlates with protein absorption and plasma creatine kinase (CK) levels, where CK serves as a biomarker for exercise-induced muscle damage. (Clarke et al., 2014). Similar findings have been confirmed in cyclists (Petersen et al., 2017) and long-distance runners (Jang et al., 2019). Unlike gut microbiota α-diversity, few studies have found correlations between exercise and gut microbiota structure (β-diversity).

This study compared gut microbiota diversity between Taekwondo and diving athletes, revealing no significant differences in either α-diversity or β-diversity among athletes from these two sport disciplines. This aligns with most current research findings; (O'Donovan et al. 2020) analyzed fecal and urine samples from 37 elite athletes across 16 sport disciplines and found no significant differences in α- or β-diversity among athletes from different sports. Additionally, (Jang et al. 2019) Comparison of gut microbiota diversity between 15 bodybuilders and 15 long-distance runners revealed no significant differences in α-diversity or β-diversity. Despite variations in exercise modalities, body composition, and dietary patterns, no significant differences in gut microbiota diversity were observed among the athletes. As the aforementioned studies were limited to cross-sectional designs, higher-quality experimental evidence is required to confirm and elucidate the underlying mechanisms.

### 4.2 Comparative analysis of gut microbiota composition among athletes from different disciplines

Gut microbiota composition varies with athletic performance levels. Elite athletes exhibit higher abundances of Firmicutes, Bacteroidetes, Proteobacteria, and Actinobacteria at the phylum level. At the genus level, Rumminococcaceae_unclassified, Clostridiales_unclassified, Faecalibacterium, and Lachnospiraceae_unclassified were enriched in adult elite athletes, while Bacteroides and Prevotella predominated in youth elite and youth non-elite athlete cohorts, respectively (Han et al., 2020). Another study involving elite international rugby athletes demonstrated that compared to control groups, athletes exhibited lower inflammatory responses and improved metabolic indicators; their gut microbiota displayed greater diversity than controls. Compared to control groups, elite athletes showed significantly higher proportions of certain taxa within their gut microbiota. However, within the athlete cohort, enhanced gut microbiota diversity was associated with exercise and dietary protein intake (Clarke et al., 2014). Notably, few studies have investigated gut microbiota variations across different athletic disciplines. Therefore, this study analyzed the gut microbiota of Taekwondo athletes and diving athletes.

The study revealed significant enrichment of Firmicutes phylum, Bacilli class, Erysipelotrichia class, Lactobacillales order, Erysipelotrichales order, Rhizobiales order, Streptococcaceae family, Erysipelotrichaceae family, Lactobacillaceae family, ^*^Lactobacillus^*^ genus, ^*^Blautia^*^ genus, ^*^Streptococcus^*^ genus, ^*^Anaerostipes^*^ genus, and Veillonellaceae bacteria in the gut microbiota of elite Taekwondo athletes. Conversely, Saccharibacteria phylum, Desulfovibrionales order, Rikenellaceae family, Desulfovibrionaceae family, ^*^Parabacteroides^*^ genus, and ^*^Lachnoclostridium^*^ genus were predominantly enriched in the intestinal microbiota of elite diving athletes. The study revealed that among the gut microbiota species enriched in Taekwondo athletes, Lactobacillaceae and the Lactobacillus genus represent the most prevalent probiotics, exhibiting multiple anti-inflammatory and antioxidant bacterial effects (Li et al., 2021). Simultaneously, they effectively improve intestinal permeability, reduce tumor necrosis factor-α (TNF-α; a pro-inflammatory marker) levels, and decrease exercise-induced protein oxidation levels (Zeng et al., 2021). Animal experiments demonstrated that mice administered Lactobacillus exhibited prolonged exhaustive swimming time, the reason being that Lactobacillus alleviates exercise-induced fatigue in mice caused by free radicals (Zeng et al., 2018). Furthermore, Lactobacillus supplementation elevates α-amylase levels *in vivo*. This enzyme primarily catalyzes starch decomposition into glucose for organism absorption, thereby reducing the incidence of gastrointestinal and urinary tract infections (Mendes et al., 2021). The Anaerostipes genus has been reported to correlate with mental fatigue. Elevated Anaerostipes levels associate with activation of fatty acid oxidation, synthesis and lipolysis inhibition, which reduce circulating lipid plasma levels and body weight. This genus also suppresses colonic inflammation and downregulates insulin signal transduction in adipose tissue (Muthuramalingam et al., 2020). Veillonellaceae are Gram-negative, anaerobic, micrococcus bacteria associated with improved HDL levels and metabolic function. Their primary role involves breaking down and utilizing lactate. By converting lactate into propionate—a short-chain fatty acid that provides energy—Veillonellaceae facilitate fatigue clearance and enhance endurance exercise capacity. Research by Scheiman et al. revealed a significant post-marathon increase in intestinal Veillonellaceae abundance among athletes. When Veillonellaceae strains from these athletes were transferred to mice, the rodents' exhaustive running time increased by 13% (Scheiman et al., 2019).

In diving athletes, the enriched Desulfovibrionales and Desulfovibrionaceae are efficient acetate-producing bacteria. Acetate, as the predominant SCFA, regulates hepatic gene expression patterns in lipid metabolism—specifically suppressing hepatic fatty acid synthase (FASN) and CD36 protein expression. This mechanism effectively treats high-fat diet-induced non-alcoholic fatty liver disease (NAFLD) in mice (Hong et al., 2021). The Rikenellaceae family, belonging to Bacteroidetes, upregulates genes involved in fatty acid oxidation within the liver through SCFA production (primarily acetate and propionate). This process inhibits adipose accumulation and prevents obesity in mice (Kondo et al., 2009). Human studies have also found that increased abundance of the Rikenellaceae phylum can reduce visceral adipose tissue in elderly individuals, ameliorate obesity, and increase lean body mass (Tavella et al., 2021). Furthermore, recent research indicates that the abundance of the Rikenellaceae phylum is highly correlated with that of the Christensenellaceae phylum. Christensenellaceae, also belonging to the Bacteroidetes phylum, produces SCFAs and shows significantly higher abundance in lean subjects compared to obese individuals (Oki et al., 2016). This may be associated with the long-term maintenance of low body weight among diving athletes.

The Parabacteroides genus has been demonstrated to convert primary bile acids into secondary bile acids (such as lithocholic acid and ursodeoxycholic acid) while generating succinate. This effectively reduces body weight, hyperglycemia, and hepatic steatosis in high-fat diet-induced obese mice, playing a crucial role in treating obesity and other lipid metabolism disorders (Wang et al., 2019). The ^*^Lachnoclostridium^*^ genus was first reported in 2016 by Amadou as a novel bacterial species isolated from the gut microbiota of healthy humans (Amadou et al., 2016), and was later found to be isolated from renal transplant patients. It belongs to Gram-positive anaerobic bacteria (Dandachi et al., 2021), which can significantly increase under 3 weeks of high-volume training intensity (Craven et al., 2022), demonstrating a close association with exercise.

### 4.3 Comparison of gut microbiota metabolic pathways among athletes from different sports

PICRUSt2 functional prediction and metabolic pathway analysis revealed 12 differential metabolic pathways between the two groups, with 7 pathways significantly higher in Taekwondo athletes than in diving athletes. The differences were primarily associated with two pathways: Glycine biosynthesis of tetrapyrrole II and myo-, chiro- and scyllo-inositol degradation. Glycine (Gly), a non-essential amino acid, plays a potential role in protecting tissues against injuries such as ischemia, hypoxia, and reperfusion (Hall, 1998). Tetrapyrroles primarily include heme, chlorophyll (Chl), and vitamin B12 (Tanaka and Tanaka, 2007). Heme, a naturally occurring iron porphyrin compound, exhibits neuroprotective effects (Yang et al., 2020), antioxidant activities (Morales et al., 2019), and the ability to induce cell apoptosis (Catalan et al., 2020). Vitamin B12 has been demonstrated to enhance the activity of methionine synthase, which converts homocysteine to methionine, thereby strengthening folic acid metabolism and reducing the incidence of neural tube defects (NTD) (Ray et al., 2002); it also shows potential therapeutic effects against cardiovascular diseases (Schwammenthal and Tanne, 2004; Stanger et al., 2003) and cancers (Ames, 2001). Thus, the tetrapyrrole metabolic pathway in glycine biosynthesis is closely associated with health outcomes. Additionally, scyllo-inositol is an endogenous stereoisomer of inositol known to inhibit the accumulation and toxicity of amyloid-β peptides and α-synuclein (McLaurin et al., 2000). Scyllo-inositol has been demonstrated to effectively degrade Htt, thereby treating the onset and progression of HD (Lai et al., 2014). This study found that Taekwondo athletes exhibited significantly higher abundances of Veillonella and Lactobacillus compared to diving athletes, with Veillonella showing associations with cognitive function in Parkinson's disease patients (Barichella et al., 2019). Lactobacillus can effectively alleviate stress and anxiety while enhancing memory and cognitive function (Lew et al., 2019). These findings indicate that the gut microbiota and metabolic pathways of Taekwondo athletes are closely related to cognitive functions and other health-related factors.

Diving athletes exhibited significantly higher activity in five metabolic pathways compared to Taekwondo athletes, with primary differences observed in Lipid IVA biosynthesis and Kdo transfer to lipid IVA III (Chlamydia). Lipid IVA activates signaling through Toll-like receptor 4 (TLR4) (Saitoh et al., 2004). TLR4 serves as an immune surveillance molecule that plays a crucial role in the body's immune defense. (Stewart et al., 2005). This study found that the abundance of ^*^Saccharibacteria^*^ and ^*^Parabacteroides^*^ genera was significantly higher in diving athletes than in Taekwondo athletes. The ^*^Saccharibacteria^*^ genus typically colonizes oral bacteria, regulating the structural hierarchy and functionality of the oral microbiome by influencing host physiological functions or directly eliminating host-associated bacteria. This modulation potentially impacts oral microbial ecology through alterations in bacterial relative abundance (Bor et al., 2019). ^*^Parabacteroides^*^ has been closely linked to the pathogenesis of multiple sclerosis (MS), an immune-mediated disease whose etiology involves both genetic and environmental factors (Chen et al., 2016). This evidence suggests that the gut microbiota and metabolic pathways of diving athletes may be closely associated with immune function. However, the reasons for the differences in gut microbiota and metabolic pathways between diving athletes and Taekwondo athletes remain unclear, and the underlying mechanisms urgently require investigation.

## 5 Conclusion

Gut microbiota analysis revealed no significant differences in gut microbiota α-diversity or β-diversity between Taekwondo and diving athletes. However, significant differences were observed in microbial species composition and metabolic pathways. The study revealed significant enrichment of Firmicutes phylum, Bacilli class, Erysipelotrichia class, Lactobacillales order, Erysipelotrichales order, Rhizobiales order, Streptococcaceae family, Erysipelotrichaceae family, Lactobacillaceae family, ^*^Lactobacillus^*^ genus, ^*^Blautia^*^ genus, ^*^Streptococcus^*^ genus, ^*^Anaerostipes^*^ genus, and Veillonellaceae bacteria in the gut microbiota of elite Taekwondo athletes. Conversely, Saccharibacteria phylum, Desulfovibrionales order, Rikenellaceae family, Desulfovibrionaceae family, ^*^Parabacteroides^*^ genus, and ^*^Lachnoclostridium^*^ genus were predominantly enriched in the intestinal microbiota of elite diving athletes. PICRUSt2 functional prediction and metabolic pathway analysis demonstrated significantly higher abundances of ^*^Veillonella^*^ and ^*^Lactobacillus^*^ in Taekwondo athletes compared to diving athletes, whereas ^*^Saccharibacteria^*^ and ^*^Parabacteroides^*^ genera were significantly more abundant in diving athletes. Taekwondo athletes and diving athletes exhibit differences in gut microbiota species composition and metabolic pathways, suggesting that gut microbiota may serve as a potential classification indicator to distinguish between these athlete groups in the future, and could provide an important basis for early-stage athlete selection.

## Data Availability

The data analyzed in this study was obtained from two elite Chinese national team athletes, and the following licenses/restrictions apply: the raw sequencing data cannot be deposited in public repositories due to Chinese national sports team management regulations and athlete privacy protection policies governing elite athletes. These restrictions are outside the authors' control, and approval for public release was not granted despite repeated coordination with the relevant national authorities. All processed data and analytical results supporting the conclusions of this study are included in the main text and supplementary materials. Raw sequencing data can be made available to qualified researchers upon reasonable formal request and the signing of a data sharing agreement that ensures compliance with athlete privacy protections and applicable regulations. Requests to access these datasets should be directed to the corresponding author.
